# Chasing Ghosts: Race, Racism, and the Future of Microbiome Research

**DOI:** 10.1128/mSystems.00604-21

**Published:** 2021-10-12

**Authors:** Travis J. De Wolfe, Mohammed Rafi Arefin, Amber Benezra, María Rebolleda Gómez

**Affiliations:** a Department of Pediatrics, University of British Columbia, Vancouver, British Columbia, Canada; b Department of Geography, University of British Columbia, Vancouver, British Columbia, Canada; c Department of Science and Technology Studies, Stevens Institute of Technology, Hoboken, New Jersey, USA; d Department of Ecology & Evolutionary Biology, University of California Irvine, Irvine, California, USA; University of Connecticut

**Keywords:** antiracist, ethnicity, justice, microbiome, microbiota, race, racism

## Abstract

In this article, we argue that a careful examination of human microbiome science’s relationship with race and racism is necessary to foster equitable social and ecological relations in the field. We point to the origins and evolution of the problematic use of race in microbiome literature by demonstrating the increased usage of race both explicitly and implicitly in and beyond the human microbiome sciences. We demonstrate how these uses limit the future of rigorous and just microbiome research. We conclude with an outline of alternative actionable ways to build a more effective, antiracist microbiome science.

## PERSPECTIVE

## INTRODUCTION: RACE AND MICROBIOME SCIENCE

We are a transdisciplinary group of researchers—a microbiologist, a geographer, an anthropologist, and an evolutionary and microbial ecologist—who are committed to antiracist scholarship and to the effective and ethical future of microbiome science. In this article, we argue that the use of race and other racial proxies as “ghost variables” in most current human microbiome research is problematic and requires interrogation. We examine how racial categories are used in the microbiome literature and conclude with alternative, actionable ways to build equitable and antiracist microbiome science.

Race has no coherent basis in biology. Human groups have extensively shared genetics over time and space through migration and forced displacement. As a result, while there are some geographic signatures of genetic variation, human populations are highly interconnected (1). There is more genetic diversity within racial categories than between “races” (2–4). Thus, genetics do not clearly map onto ideas of race. Racial categories vary across cultural and historical contexts and are socially determined (5). Nevertheless, the harmful effects of historical and contemporary racism have real impacts on people’s lives, bodies, and environments (6). As Amutah et al. argue, “Race is not a biologic category based on innate differences that produce unequal health outcomes. Rather, it is a social category that reflects the impact of unequal social experiences on health” (7). These uneven experiences must be studied in relation to the disease, environmental, and socioeconomic burdens of Black, Indigenous, and People of Color (BIPOC). Categories of race in the microbiome sciences must be used intentionally and with care, and race must be studied in relation to racism.

The microbiome sciences are often complicit in contributing to racial disparities by attributing findings to racial or ethnic differences without referencing racism or by using ghost variables of race. By ghost variables, we are referring to complex, historically loaded racial categories used in microbiome research without explicitly naming race (8). Studies use imprecise labels that inaccurately conflate race with other variables, present racial or ethnic differences in disease or environmental burden without context, or link racial groups with particular diseases or increased disease burden (6). In continuing to use the category of race as a determining variable in research design and analysis, human microbiome research has come to explicitly or implicitly rely on race as holding biological truth independent of social forces. Race, as Kozik demonstrates, serves as a “conveniently measurable proxy” without attending to the confounding structure of racism and its many effects on people and environments (9, 10).

In this article, we combine Ruth Wilson Gilmore’s definition of racism, the production and exploitation of “group-differentiated vulnerability to premature death,” with Paul Farmer’s use of structural violence, the normalized, interacting political and social structures that cause injury, injustice, and oppression, to examine race in studies of the microbiome (11, 12). If the microbiome sciences continue to explicitly and implicitly deploy the variable of race in research without accounting for the relevant forms of racism that impact people and environments, microbiome research will continually provide data and recommendations in support of a system based on inequities and harm.

## RACE IN STUDIES OF THE HUMAN MICROBIOME

How did race come to occupy an important role in microbiome research? The advancement of genomics along with the initial findings of the Human Genome Project stoked enthusiasm for personalized medicine and the idea of a postracial science. Findings suggest that humans are more genetically similar than historically assumed, making biological justifications for racial categories untenable. Yet, biological understandings of race reemerged in studies of genetic ancestry (13–15). For the microbiome sciences, this resurgence exerted itself in subtle but powerful ways. For example, a primary goal of the Human Microbiome Project was to characterize the “healthy” microbiome as a critical first step in determining how deviation from a baseline state contributes to human disease (16). From this early work, major innovations included new sequence databases, laboratory methods and technologies, and bioinformatic tools. These advancements triggered a deluge of correlative microbiome studies in and beyond the biomedical sciences. While there have been advancements in mechanistic understandings of the microbiome, the definition of a “healthy microbiome” remains ambiguous (17). In this context characterized by uncertainty, race emerged as a common variable used to make deterministic and comparative claims about the microbiome and to explain health disparities among racial or ethnic groups (8, 18–21).

**FIG 1:**
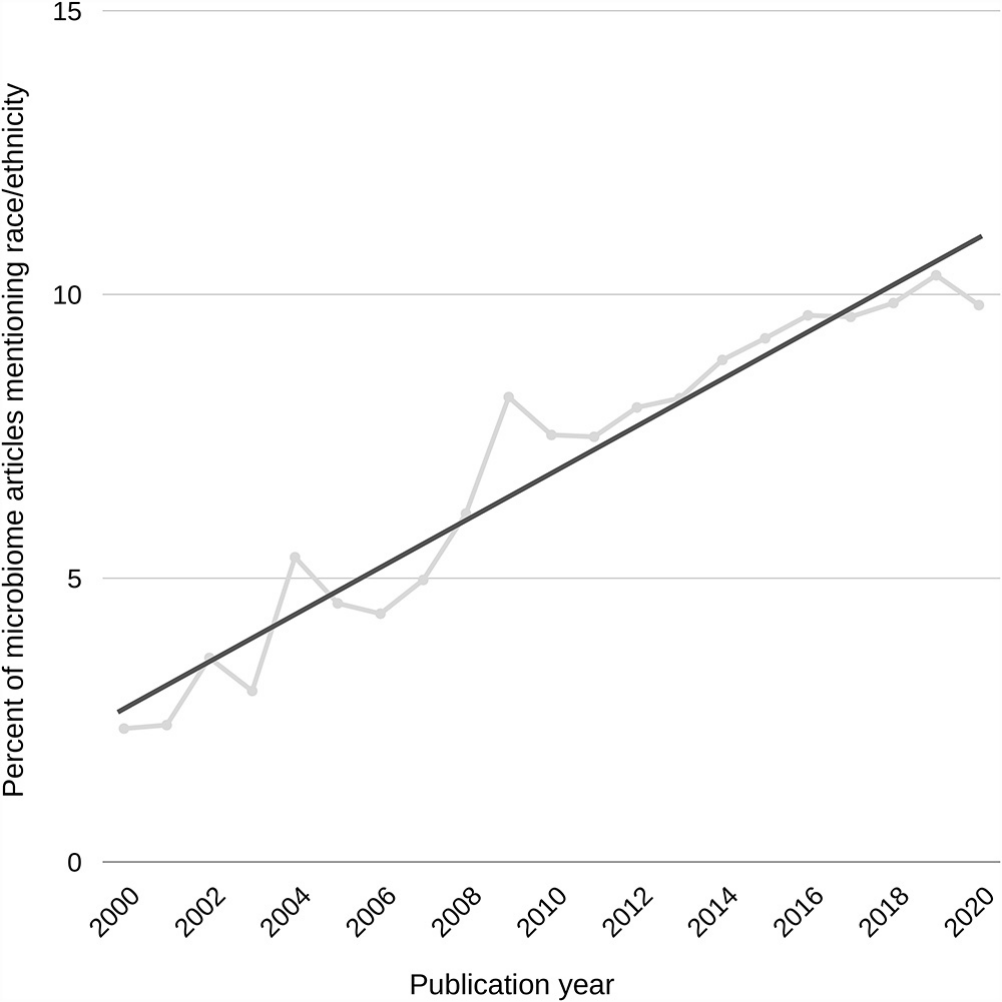
The use of race/ethnicity among human microbiome research has steadily increased since 2000. Data represent Boolean search results of the PMC archive using *rentrez* in R (69, 70). Search terms representing the human microbiome included microbiome, microbiota, or 16S rRNA and human, patient, subject, volunteer, or participant. Search terms representing race or ethnicity included race, racial, or ethnic.

Among the human microbiome literature archived in PubMed Central (PMC) between 2000 and 2020, there are 14,103 results that mention race or ethnicity. Representative examples of this type of widely cited scholarship describe race as one of the strongest host phenotypes associated with the microbiome (19, 22–24). While a growing body of peer-reviewed human microbiome research exists mentioning race or ethnicity (Fig. 1), it is worrisome that only 114 of the 14,103 PMC-archived human microbiome articles also mention racism. Further review of those results reveals that only 8 of the 114 articles have engaged with the specificity of how racism is embedded in microbiome research; many of them are rarely cited (9, 25–31). These results suggest that current microbiome research risks using race or ethnicity as an explanatory factor—determinant or correlative—of the microbiome. Therefore, the field has limited engagement with the direct effects of racism on human physiology, which include but are not limited to increased trauma and stress, lack of access to quality health care, and historical and current discrimination in treatment within health care systems.

## RACE AS A GHOST VARIABLE IN AND BEYOND THE HUMAN MICROBIOME

**FIG 2:**
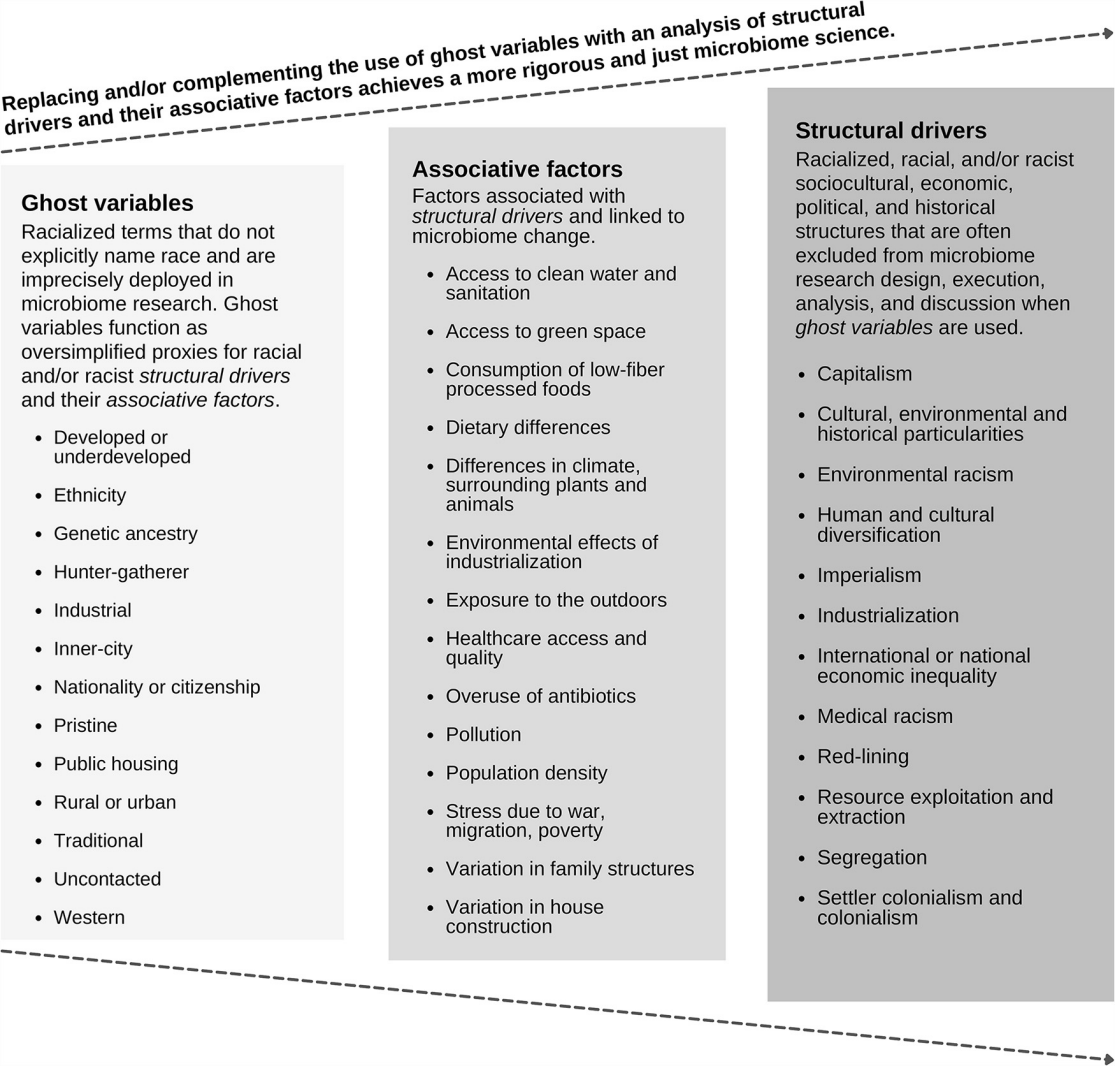
Replacing and/or complementing the use of ghost variables with an analysis of structural drivers and their associative factors achieves a more rigorous and equitable microbiome science. This figure is not exhaustive but is intended to assist researchers in determining study groups during the early stages of research design or to help analyze existing studies’ use of race or ghost variables.

With increased recognition of the problems associated with using race as a category of analysis in microbiome science, we must also examine how often race is used as a ghost variable. Human microbiomes are categorized as “Western,” “industrialized,” or belonging to “Europeans” and “Americans” and compared against the microbiomes of racialized Indigenous and global South populations whose microbiomes are presupposed to be “underdeveloped,” “modernizing,” and closer to “pure” or “natural” states (32–36). Assuming that the microbiomes of Indigenous groups are a baseline somehow ancestral to all humans is a notion that ignores the complexity of genetic, ecological, and cultural divergence in human populations and fails to account for the rapid ecological and evolutionary changes in the microbiome (Fig. 2).

Throughout this kind of microbiome research is the assertion that BIPOC populations are less developed and have “wild” or “natural” microbiota and that “modern,” “disturbed,” “Western/white” microbiomes must be “rewilded.” In some of these studies, racial categories are implemented to address differences in health outcomes, but they fail to acknowledge social history and complexity, or the ecological intricacy of the microbiome itself. For example, studies looking at racial differences in vaginal microbiomes briefly consider correlated socioeconomic differences, but they do not account for the effects of stress and toxic exposures associated with structural racism (18, 20). Furthermore, this work fails to address BIPOC medical distrust and how that affects reporting and data. Similarly, studies of the effects of migration on the human microbiome often look at immigration to “Western” and “industrialized” countries but rarely at migration in the opposite direction to understand the effects of the new environment and diet (37).

Race as a ghost variable also extends beyond the human microbiome. In studies of the microbiome of the built environment, spaces are racialized but analyzed without explicit mention of race or structural racism. Studies investigate urban built environments and draw connections between microbiota and higher rates of asthma, inflammatory bowel diseases, and even affective and anxiety disorders—but race or ethnicity is rarely mentioned (38, 39). The omission of race elides effects of structural racism such as histories of segregation, red-lining, and enduring environmental injustice that creates spatial divisions in cities. Furthermore, in environmental microbiome studies, landscapes that have been long tended by Indigenous populations are often described as “pristine” or “undisturbed” (40–42), erasing the contributions of Indigenous peoples to these ecosystems and microbiomes (43, 44).

This article is focused on how race is used as an explicit or implicit category in microbiome research. But race is a “ghost” in other important ways—a lack of diversity and representation in researchers and study populations and a lack of meaningful community research engagement prevent important questions from being asked.

## CONCLUSION: TOWARD AN ANTIRACIST MICROBIOME SCIENCE

We have traced how race in many iterations has been operationalized in microbiome science. Historically and presently, microbiome sciences either harmfully ignore systemic racism and its effects or unreflectively reproduce racial thinking—with far-reaching implications for the study of human, environmental, animal, and plant microbiota.

From the global pandemic to uprisings against racial injustice, this is a transformative moment. Microbiome researchers have the opportunity to make significant changes to the ways science addresses race and simultaneously improve the quality and precision of this important work. An emerging literature is calling for microbiome science to address racial disparities (8, 45, 46), and increasingly, human microbiome studies are directly addressing socioenvironmental aspects causing differences in microbiome composition and health outcomes (47–49). Unlike race or ethnicity, there are clear mechanisms linking these variables with microbiome composition; interventions on these environmental variables are possible and can directly address environmental and health inequalities. However, work must be done across microbiome science to connect differences in microbiota to health disparities caused by structural inequities (9, 50, 51).

We propose an integrated three-part approach to create antiracist microbiome science. We build upon work in Indigenous science and technology studies and literature on race and genomics to suggest actionable solutions for microbiome science (52–61). First, institutional changes must be made in funding, publishing, hiring, and recruiting practices. Second, transdisciplinary collaboration across the biological and social sciences must be established as essential and customary. Third, study populations and BIPOC communities must be engaged in the research and empowered through the science. These suggestions are ordered by their relative feasibility: we see the first as the most feasible and the third as the most challenging to implement but most impactful.

1. STEM fields and microbiome sciences in particular continue to lack representational diversity (62, 63). BIPOC, people with disabilities, and gender-diverse students and scientists should be sought out and supported for academic research, teaching jobs, funding, and publication. Publishing and funding work by researchers from different backgrounds reduce bias and prevent omissions in data (64). Funders and journal reviewers must be diversified and can be trained to look critically at how concepts of race are being utilized in proposed work. We suggest that funding agencies and editors pledge to interrogate papers and grant proposals that use race without accounting for or referencing racism. This is the future gold standard for educating junior scientists, and thoughtful antiracist thinking should be a requirement for funding and publishing.
2. As we endeavor to challenge racism, we assert that social science-microbiome science partnerships are central to this work. We use “transdisciplinary” to highlight the need to work across disciplines, types of knowledge, and expertise, integrating natural, social, and health sciences and transcending the boundaries of those fields (65). Anthropologists, geographers, social scientists, epidemiologists, and public health experts can contribute to the analysis of variables that have real explanatory power and can examine the ways sociopolitical systems interact with the microbial world, thus enriching and improving the science. Ultimately, scientific research can no longer in good faith use race as an inadequate and misleading proxy. More precise variables (such as food, environment, infrastructure, social relationships, and structural racism) need to be studied. Race is not a valid biological category, but racism has a consequential effect on biology.
3. As mentioned above, microbiome science has failed to attend to an “ethics of care” in regard to marginalized people and environments (66). To work toward antiracist research, we propose that microbiome researchers look to current science that intentionally engages with the problem of racism (please see work by the following: Dr. Rosie Alegado, University of Hawaiʻi at Mānoa; Dr. Katherine Amato, Northwestern University; Dr. Marie-Claire Arrieta, University of Calgary; Dr. Erin Eggleston, Middlebury College; Dr. Keolu Fox, UC San Diego; Dr. Sue Ishaq, University of Maine; Dr. Michael D. L. Johnson, University of Arizona; Drs. Cecil M. Lewis, Jr., and Paul Spicer, University of Oklahoma; Dr. Max Liboiron, Memorial University; Dr. Kat Milligan-Myhre, University of Connecticut) and consider the following conditions when working with BIPOC subjects and communities:
  - Prioritize community engagement and community-led research that utilizes local knowledge in research design from the conception of each project.
  - Solicit community and individual input on health and environmental priorities.
  - Secure formal consent from each community/tribe/sovereignty/nation for sampling, land use, and community access.
  - Participate in socially responsible sampling, management, and fair ownership of data (67).
  - Actively account for social determinants of human and environmental health in scientific data, which could include systemic barriers like poor access to health care, jobs, housing, and education.
  - Ensure equitable benefit-sharing of translational interventions developed from the scientific research for research participants and their communities.
  - Legally protect subjects, their samples, data, and land against commercial, scientific, medical, and cultural exploitation (68).

We are very aware that what we are proposing is extremely difficult and that the practices we have outlined are not currently supported by funding structures, study designs, or institutional hierarchies. Which is precisely why, for instance, future human microbiome grants must add field-inclusive funding strategies that support equity initiatives. We challenge microbiome researchers using racial categories to ask themselves, what is the function of race in my study? What am I using it for, and is there something more precise and equitable (Fig. 2)? This article is just the barest of beginnings; developing an antiracist microbiome science requires investment by all to determine what the field standards should be and how to deploy all types of expertise and knowledge systems to address systemic drivers of microbial difference. Very few have started to put this work into action yet, but we have cited some researchers who can serve as methodological inspirations. There is no guidebook, but with determined commitments to equity, collaboration, and better science, it is a goal worth striving toward.
